# Comprehensive Phytochemical Analysis of Various Solvent Extracts of *Artemisia judaica* and Their Potential Anticancer and Antimicrobial Activities

**DOI:** 10.3390/life12111885

**Published:** 2022-11-14

**Authors:** Merajuddin Khan, Mujeeb Khan, Khaleel Al-hamoud, Syed Farooq Adil, Mohammed Rafi Shaik, Hamad Z. Alkhathlan

**Affiliations:** Department of Chemistry, College of Science, King Saud University, P.O. Box 2455, Riyadh 11451, Saudi Arabia

**Keywords:** terpenes, volatiles, GC–MS, biological activities, phytoconstituents

## Abstract

Solvents play an important role in the extraction process by considerably affecting the amount and nature of secondary metabolites of medicinal plants. Thus, the effect of solvents must be investigated to obtain desired biological properties of plant extracts. In the current study, we extracted aerial parts of *Artemisia judaica,* native to Saudi Arabia, in three different solvents, including methanol (MeOH), hexane (Hex), and chloroform (Chl). Obtained extracts from the aerial parts of *A. judaica* were analysed by GC–MS and GC–FID techniques, which resulted in the identification of 46, 18, and 17 phytoconstituents from the Hex, Chl, and MeOH extracts, respectively. All the extracts contain oxygenated terpenes, aliphatic hydrocarbons, and aromatics as major classes of compounds in varying amounts. Among the various phytoconstituents identified, piperitone was the dominant compound and was found in all the extracts in different amounts, specifically, 28.8, 26.1, and 20.1% in the Chl, MeOH, and Hex extracts, respectively. Moreover, all these extracts (Chl, MeOH, and Hex) were tested for the antimicrobial properties on both Gram-positive and negative bacteria as well as for their anticancer properties on four different cell lines including HepG2, DU145, Hela, and A549. Among the different extracts, the Hex and Chl extracts demonstrated identical antimicrobial properties, while the Chl extract showed superior anticancer properties when compare to the other extracts. The higher biological properties of Chl extracts including both antimicrobial and anticancer activities may be attributed to the presence of large amounts of piperitone and/or santonin, which are distinctly present in excess amounts in the Chl extract.

## 1. Introduction

Plants are an important source of several pharmaceuticals that are currently used as therapeutics for pain (e.g., morphine); various diseases, including cancer (e.g., vincristine); bacterial and fungal infections (e.g., penicillin); and several heart diseases (e.g., warfarin) [1]. Particularly, in the underdeveloped regions of the world where essential health services are not easily available, plant-based traditional medicines have been proven as life-saving resources [2]. Plants offer extraordinary chemical diversity and excellent capability of producing highly complex novel phytomolecules with varying chemical functionalities [3]. Plants contain a variety of secondary metabolites with diverse properties that are responsible for major organoleptic characteristics of plant-derived foods and beverages, which offer great medical or health benefits. These types of food products and supplements are often referred as “nutraceuticals”, which are extensively used in the prevention and treatment of several diseases. In this regard, the functional properties of various plant extracts are being extensively investigated for their use as novel nutraceuticals and functional foods [4,5]. Despite the tremendous potential of plants in modern medicine, among an estimated 350,000 known vascular plant species, a large number of plants still has to be chemically explored for the purpose of drug discovery [6]. However, to date, the discovery of therapeutic phytomolecules still remains challenging due to various legal and logistical hassles in the exploration and procurement of medicinal plants [7]. Moreover, the processes of bioassay-guided fractionation and isolation of active phytomolecules are both cumbersome and costly, which often deters the pharmaceutical industry and government agencies from perusing medicinal plant-based research programs [8].

The discovery of therapeutically active phytoconstituents begins with the exploration of medicinal plants and the extraction of bioactive compounds from plant materials [9]. So far, significant progress has been made in the processes of extraction, purification, and isolation of activity-guided bioactive compounds [10]. Among various methods, conventional solvent extractions have been commonly applied to produce the plant extracts due to their ease of use, efficiency, and wide applicability [11]. Plants extracts are typically prepared with a variety of solvents that are known to produce different types of phytomolecules depending on the difference in polarity of the solvents [12]. For instance, polar solvents are typically used to extract phenolic components and their glycosidic derivatives, saponins, etc., whereas fatty acids, steroids, etc. are extracted using non-polar solvents [13]. Indeed, several studies have reported the effect of solvents on the variety of secondary metabolites and/or their biological properties [14]. Therefore, to enhance the biological properties of phytoconstituents, proper selection of extraction solvents and extraction techniques are highly required. To achieve this, comparative biological studies of same plant extract extracted from different solvents are beneficial. For example, Syukriah et al. identified water as the highest producer of bioactive constituents of the *Quercus infectoria* (manjakani) plant, which was extracted from six different solvents [15]. However, only a smaller number of similar studies have been performed so far on Saudi medicinal plants.

**Figure 1:**
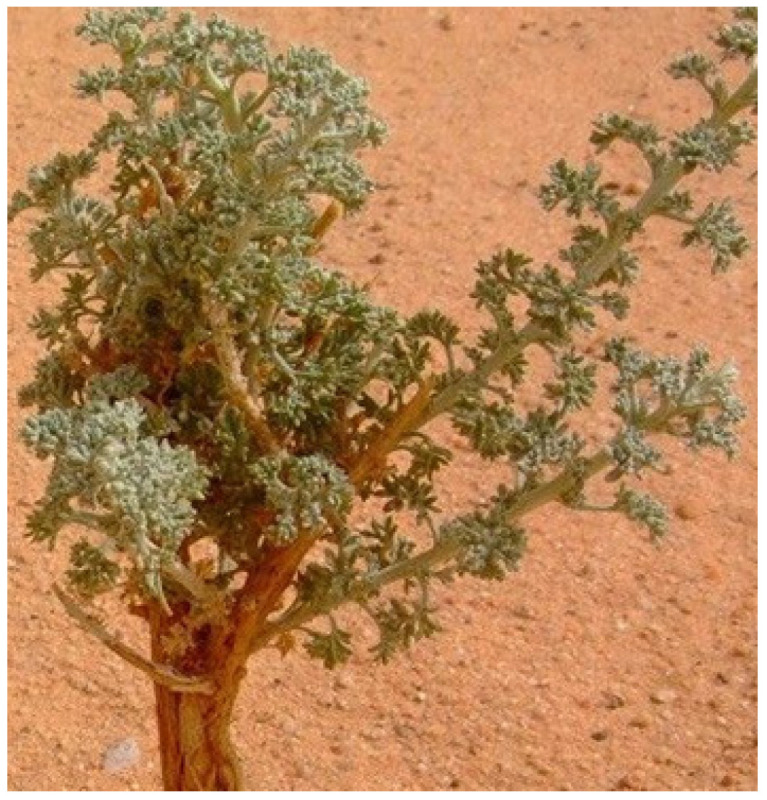
*A. judaica* in its natural habitats.

*Artemisia* is an important genus belonging to the Asteraceae family. Several species of the genus Artemisia have been potentially used as important sources of nutraceuticals [16]. Among various Saudi medicinal plants, *Artemisia judaica* L. (*A. judaica*) has long been used to treat several ailments, including cardiovascular diseases, skin disorders, cancer, arthritis, immune deficiencies, etc. [17]. Several studies have been reported so far on the biological importance of *A. judaica* of Saudi Arabia; for instance, the volatile oil contents of *A. judaica* grown in the northern region of Saudi Arabia have demonstrated the presence of a variety of phytoconstituents that have shown decent antimicrobial properties [18]. In another study, the volatile chemical constituents of *A. judaica* from the central region of Saudi Arabia revealed the presence of a different class of compounds from the plant volatile oils when explored using a combination of gas chromatography techniques [19]. These phytoconstituents have exhibited admirable antibacterial properties. However, to the best of our knowledge, the extracts of *A. judaica* grown in the western part of Saudi Arabia have not been explored yet for their bioactive constituents and biological properties. *A. judaica* L. (Figure 1) is a small shrub with pubescent leaves and a perennial fragrance that grows widely in Saudi Arabia. It is considered as a rich source of flavonoids including apigenin, cirsimaritin, and various other compounds like camphor, piperitone, 1,8-cineole, chrysanthenone, thujones, etc. [20]. To date, several previous studies largely focused on the screening of phytoconstituents and/or biological activities of the volatile components of *A. judaica*. However, there is no detailed report on the phytoconstituents of *A. judaica* extracted using different polarities of solvents and comparisons of their biological activities including anticancer and antimicrobial properties. Thus, in this study, our main aim was to investigate the phytochemical constituents of *A. judaica* extracted from different solvents and their anticancer and antimicrobial properties. For this purpose, the aerial parts of the plant were extracted using three different solvents such as hexane (Hex), chloroform (Chl), and methanol (MeOH). Each plant extract of *A. judaica* was analysed separately to determine their chemical constituents and to assess their biological properties.

## 2. Materials and Methods

### 2.1. Plant Material

Entire aerial parts of *A. judaica* grown in the region of Madinah, a city in the Western part of Saudi Arabia, were procured in April 2020. Identifications of *A. judaica* were authenticated by Dr. Rajakrishnan Rajagopal from the herbarium division of King Saud University. A specimen sample (AJMED-21) of *A. judaica* is retained with us.

### 2.2. Chemicals

All the chemicals including methanol, chloroform, and *n*-hexane were of analytical grade and purchased from Sigma–Aldrich, Germany. Pure volatile constituents or enriched fractions of volatile constituents such as camphene (Sigma–Aldrich, Burlington, MA, USA), heptacosane, carvacrol (Sigma Aldrich, Shanghai, China), thymol (Alfa Aesar, Lancashire, UK), piperitone, caryophyllene oxide, and spathulenol (enriched fractions) were available and used for co-injection/comparative analysis.

### 2.3. Preparation of A. judaica Extracts

**Figure 2:**
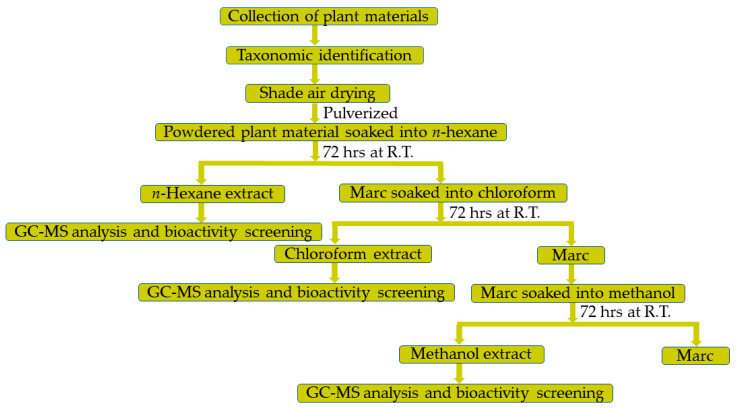
Flowchart for the preparation of *A. judaica* extracts and screening of their bioactivity.

Procured *A. judaica* plant materials were air-dried at room temperature until constant weight was achieved. The dried plant material was then grounded to a suitable mesh size using a grinder. Obtained plant material (250 g) were first percolated with *n*-hexane (500 mL) three times at room temperature. After *n*-hexane extraction, the marc was again subjected to extraction three times with CHCl_3_ (500 mL). Finally, the process of extraction was repeated using the residual marc with methanol (500 mL) for three more times at room temperature. Notably, each time, the extraction process was carried out for 3 days for all the solvents used. The resultant *n*-hexane, chloroform, and methanol extracts were separately dried under vacuum at 40 °C until solvents were completely removed using a Buchi rotary evaporating system (Rotavapor R-215, Buchi, Flawil, Switzerland) equipped with vacuum controller (V-850) and vacuum pump (V-700). These separately dried *n*-hexane, CHCl_3_, and methanol extracts were used for the screening of anticancer and antimicrobial activities and for GC analysis (Figure 2).

### 2.4. GC and GC–MS Analysis of A. judaica Extracts

In order to identify the chemical constituents of the extracts of *A. judaica*, dried extracts, i.e., n-hexane and CHCl_3_ extracts were dissolved in diethylether, whereas methanol extract was dissolved in methanol and subjected to GC–FID and GC–MS analyses. The GC–MS system was equipped with stationary phase columns (HP-5MS) employing the same method as described previously [21]. Detailed methodology is given in Supplementary Materials (Scheme S1). The identified constituents from CHCl_3_, *n*-hexane, and methanol extracts of *A. judaica* and their relative percentages are provided in Table 1 and the constituents are listed according to their elution order on the HP-5MS column.

### 2.5. Calculation of Linear Retention Indices (LRIs)

LRI values of chemical constituents of *A. judaica* extracts were determined following a previously reported method [21], and they are listed in Table 1. Detailed methodology is provided in Supplementary Materials (Scheme S2). 

### 2.6. Identification of Volatile Components

**Figure 3:**
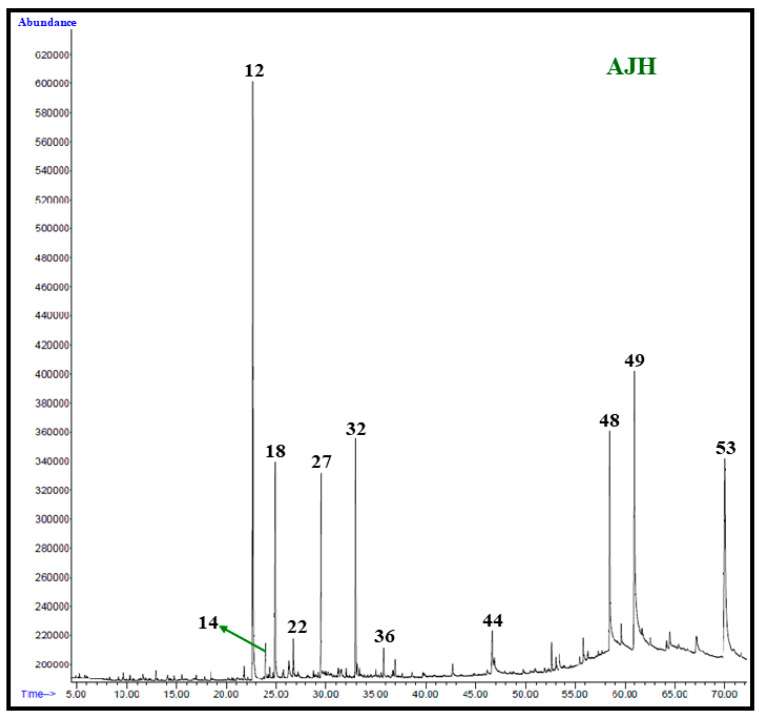

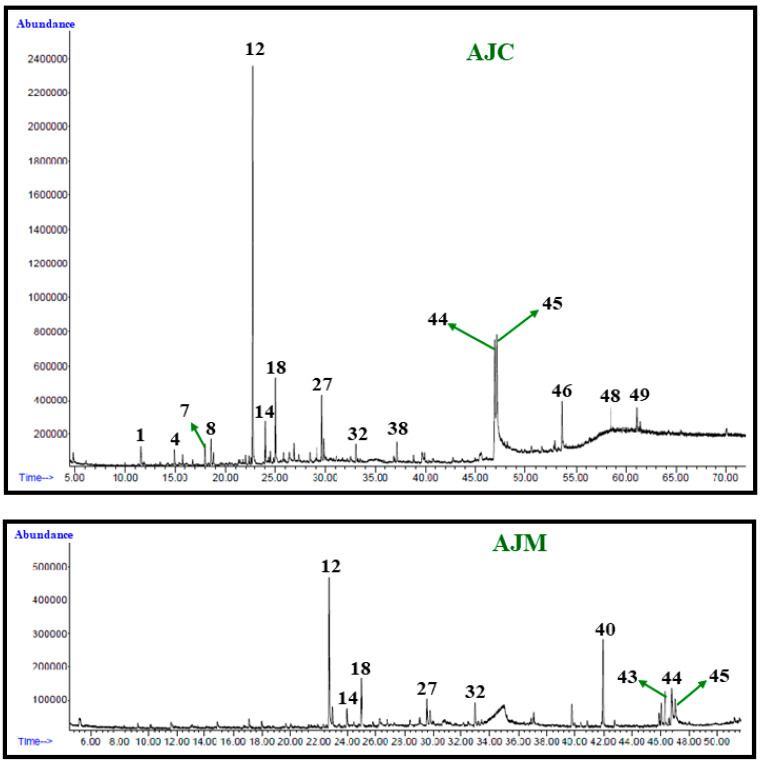
GC–MS chromatograms of *n*-hexane (**AJH**), chloroform (**AJC**), and methanol (**AJM**) extracts of *A. judaica*.

Identification of the chemical constituents of *A. judaica* extracts were carried out through analysis on a HP-5MS column as described previously [21]. Detailed methodology is provided in Supplementary Materials (Scheme S3) [22,23,24]. GC–MS chromatograms for the identified constituents of *n*-hexane, chloroform, and methanol extracts of *A. judaica* on HP-5MS column are given in Figure 3.

### 2.7. Evaluation of Antimicrobial and Anticancer Activity

#### 2.7.1. Antimicrobial Activity

Antimicrobial activity of the *A. judaica* extracts was examined using the well diffusion method [25] towards a panel of four pathogenic bacterial strains, including *Staphylococcus aureus* MTCC 96, *Micrococcus luteus* MTCC 2470, *Escherichia coli* MTCC 739, and *Klebsiella planticola* MTCC 530. The four pathogenic reference strains were spread on the surface of Mueller–Hinton agar Petri plates with 0.1 mL of previously prepared microbial suspensions individually containing 1.0 × 10^7^ CFU/mL (equal to 0.5 McFarland standard). Using a cork borer, wells of 6.0 mm diameter were prepared in the media plates, and the prepared test extracts at a dosage range of 250–0.48 µg/well were added in each well under sterile conditions in a laminar air flow chamber. Standard antibiotic solution of Ciprofloxacin at a dose range of 250–0.48 µg/well and the well containing dimethyl sulfoxide (DMSO) served as positive and negative controls, respectively. The plates were incubated for 24 h at 37 °C, and the well containing the least concentration showing the inhibition zone was considered as the minimum inhibitory concentration (MIC). All experiments were carried out in duplicates and mean values are represented.

#### 2.7.2. Anticancer Activity

Cytotoxicity of test extracts was assessed against the human lung adenocarcinoma cell line (A549), human hepatocarcinoma cell line (HepG2), human cervical cancer cell line (HeLa), and human prostate cancer cell line (DU145) using MTT assay [26]. Briefly, 1 × 10^4^ exponentially growing cells were seeded into each 96-well plate (counted by Trypan blue exclusion dye method) and allowed to grow until 60–70% confluence, then different concentrations of test extracts were added to the culture medium along with negative (DMSO) and positive controls (Doxorubicin). The plates were incubated for 48 h in a CO_2_ incubator at 37 °C with a 90% humidified atmosphere and 5% CO_2_. Then, the media of the wells were replaced with 90 µL of fresh serum-free media and 10 µL of MTT (5 mg/mL of PBS), and the plates were incubated at 37 °C for 2 h. The media was discarded and allowed to dry for 30 min. Later, 100 µL of DMSO was added in each well to dissolve the purple formazan crystals and the absorbance was recorded at 570 nm using Spectra Max plus 384 UV-Visible plate reader (Molecular Devices, Sunnyvale, CA, USA). Each test compound was examined at various concentrations in triplicate and the results are expressed as mean with standard deviation (mean ± SD), (*n = 3*). One-way ANOVA and Dunnett’s post-comparison test were used to analyse the data for significant differences (test vs. control). The statistical significance for the experiment was set at *p* < 0.05.

## 3. Results and Discussion

Herein, our aim was to explore the variability of phytoconstituents of the aerial parts of *A. judaica* using three different extraction solvents including polar, medium-polar, and non-polar solvents of methanol (MeOH), chloroform (Chl), and hexane (Hex), respectively. In addition, the evaluation of the biological properties including the antibacterial and anticancer activities of these three extracts was also performed. After complete drying and extraction of the samples, the amounts of resultant extracts from different solvents were measured. The extraction process was initiated with 250 g of aerial parts of *A. judaica* in each solvent, which yielded 4.1 g, 4.4 g, and 4.8 g of plant extract in hexane, chloroform and MeOH, respectively. Notably, different solvents resulted in the variable extract yields, which can be attributed to the nature and quantity of secondary metabolites extracted. In this case, the MeOH extract had the highest yield, which may be due to the higher solubility of polar carbohydrates and glycosides of secondary metabolites in the methanolic solution. The phytochemical analyses of the samples were performed by GC–MS and GC–FID techniques which led to the identification of a total of 46, 18, and 17 chemical constituents from the Hex, Chl, and MeOH extracts, respectively (Figure 3). All the identified phytoconstituents obtained from the three extracts and their respective proportions are given in the Table 1 according to their elution order on the HP-5MS column.

**Table 1:** Chemical constituents identified from the different solvent extracts of *A. judaica* aerial parts.

Peak	Compound *	M.F.	CAS No.	R.T. (min)	LRI_Lit_	LRI_Exp_	Hex %	Chl %	MeOH %
1	Camphene	C_10_H_16_	79-92-5	11.501	946	953	0.356	1.632	-
2	Mesitylene	C_9_H_12_	108-67-8	13.051	994	994	0.17	-	-
3	Undecane	C_11_H_24_	1120-21-4	17.083	1100	1100	-	-	1.223
4	Lavender lactone	C_7_H_10_O_2_	1073-11-6	14.854	1034	1041	0.492	1.138	-
5	Artemisia ketone	C_10_H_16_O	546-49-6	15.677	1056	1062	0.254	-	-
6	*p*-Cymenene	C_10_H_12_	1195-32-0	16.722	1089	1089	0.265	-	-
7	Isophorone	C_9_H_14_O	78-59-1	17.92	1118	1122	0.731	1.702	-
8	*p*-Menth-2-en-1-ol	C_10_H_18_O	29803-81-4	18.526	1136	1138	0.419	2.01	-
9	4-Oxoisophorone	C_9_H_12_O_2_	1125-21-9	18.764	1142	1144	0.297	-	-
10	Nordavanone	C_11_H_18_O_2_	54933-91-4	21.902	1231	1232	0.343	-	-
11	Cuminaldehyde	C_10_H_12_O	122-03-2	22.325	1242	1244	0.324	-	-
**12**	**Piperitone**	**C_10_H_16_O**	**89-81-6**	**22.797**	**1249**	**1258**	**20.154**	**28.846**	**26.154**
13	(2*E*)-Decenal	C_10_H_18_O	3913-81-3	22.968	1260	1263	-	-	3.183
14	Thymol	C_10_H_14_O	89-83-8	24.003	1289	1293	2.194	3.507	2.889
15	Carvacrol	C_10_H_14_O	499-75-2	24.328	1298	1303	0.437	-	-
16	*cis*-Methyl cinnamate	C_10_H_10_O_2_	19713-73-6	24.486	1299	1307	0.714	-	-
**17**	Filifolide-A	C_10_H_14_O_2_	50585-61-0	24.806	1318	1317	0.156	-	-
18	**Myrtenyl acetate**	**C_12_H_18_O_2_**	**1079-01-2**	**25.011**	**1324**	**1324**	**6.722**	**7.536**	**7.83**
19	Piperitenone	C_10_H_14_O	491-09-8	25.711	1340	1345	0.166	-	-
20	Ethyldihydrocinnamate	C_11_H_14_O_2_	2021-28-5	25.792	1347	1348	0.527	-	-
21	*cis*-Carvyl acetate	C_12_H_18_O_2_	1205-42-1	26.389	1365	1366	0.235	-	1.132
22	*cis*-Ethylcinnamate	C_11_H_12_O_2_	4610-69-9	26.811	1376	1379	2.402	1.331	-
23	*trans*-Methylcinnamate	C_10_H_10_O_2_	1754-62-7	27.038	1376	1386	0.12	-	-
24	*β*-caryophyllene	C_15_H_24_	87-44-5	28.368	1417	1428	0.115	-	-
25	Aromadendrene	C_15_H_24_	109119-91-7	28.889	1439	1445	0.103	-	-
**26**	Seychellene	C_15_H_24_	20085-93-2	29.07	1444	1451	0.431	1.101	-
27	***trans*** **-Ethylcinnamate**	**C_11_H_12_O_2_**	**103-36-6**	**29.606**	**1465**	**1469**	**6.325**	**5.214**	**4.629**
28	*γ*-Gurjunene	C_15_H_24_	22567-17-5	29.824	1475	1476	-	1.978	2.859
29	Myristicin	C_11_H_12_O_3_	607-91-0	31.308	1517	1526	0.706	-	-
**30**	5,6,7,7a-Tetrahydro-4,4,7a-trimethyl-2(4H)-benzofuranone	C_11_H_16_O_2_	15356-74-8	31.616	1535	1536	0.248	-	-
31	Artedouglasia oxide-A	C_15_H_22_O_3_	115403-96-8	31.72	1534	1540	0.169	-	-
32	**Spathulenol**	**C_15_H_24_O**	**6750-60-3**	**33.034**	**1577**	**1585**	**5.09**	**1.632**	**3.361**
33	Caryophyllene oxide	C_15_H_24_O	1139-30-6	33.224	1582	1592	0.403	-	-
34	Allyltetramethoxybenzene	C_13_H_18_O_4_	15361-99-6	33.483	1603	1600	0.48	-	-
35	*γ*-Dodecalactone	C_12_H_22_O_2_	2305-05-7	35.606	1676	1678	0.184	-	-
36	Apiol	C_12_H_14_O_4_	523-80-8	35.863	1677	1687	1.3	-	-
37	Nonyl phenol	C_15_H_24_O	25154-52-3	36.911	1727	1726	0.188	-	-
38	(1*E*)-1-Ethylidene-7a-methyloc tahydro-1H-indene ^a^	C_12_H_20_	56324-69-7	37.122	-	1734	1.123	1.696	2.013
39	7-Hydroxycoumarin	C_9_H_6_O_3_	93-35-6	39.844	1836	1840	0.203	-	3.875
40	**Methyl hexadecanoate**	**C_17_H_34_O_2_**	**112-39-0**	**41.949**	**1921**	**1925**	**-**	**-**	**13.522**
41	2-[(1,3-Dimethyl-1H-pyrazol-4-yl)methylene]-3,4-dihydro-1-(2H)naphthalenone ^a^	C_16_H_16_N_2_O	999476-23-5	45.88	-	2090	-	-	2.444
**42**	Heneicosane	C_21_H_44_	629-94-7	46.029	2100	2100	-	-	3.975
**43**	**Methyl linoleate**	**C_19_H_34_O_2_**	**112-63-0**	**46.291**	**2095**	**2107**	**-**	**-**	**6.13**
**44**	***α*** **-Santonin**	**C_15_H_18_O_3_**	**481-06-1**	**46.82**	**2117**	**2129**	**1.758**	**13.715**	**7.769**
**45**	***β*** **-Santonin**	**C_15_H_18_O_3_**	**481-07-2**	**47.022**	**-**	**2138**	**0.559**	**17.157**	**5.011**
46	Methyl 9,10-methylene-hexadecanoate ^a^	C_18_H_34_O_2_	1000336-51-3	53.607	-	2413	0.299	3.415	-
**47**	Pentacosane	C_25_H_52_	629-99-2	55.946	2500	2500	0.243	-	-
**48**	**Hexacosane**	**C_26_H_54_**	**630-01-3**	**58.529**	**2600**	**2600**	**9.52**	1.37	-
**49**	**Heptacosane**	**C_27_H_56_**	**593-49-7**	**61.123**	**2700**	**2700**	**13.973**	1.825	-
50	Octacosane	C_28_H_58_	630-02-4	62.711	2800	2800	0.355	-	-
51	Nonacosane	C_29_H_60_	630-03-5	64.648	2900	2900	0.91	-	-
52	Triacontane	C_30_H_62_	638-68-6	67.233	3000	3000	0.536	-	-
**53**	**9,19-Cyclo-9*β*-lanost-24-en-3*β*-ol, acetate ^a^**	**C_32_H_52_O_2_**	**1259-10-5**	**70.165**	**-**	**3106**	**12.106**	-	-
*Monoterpenes hydrocarbons*							0.621	1.632	-
*Oxygenated monoterpenes*							29.004	42.899	39.005
*Sesquiterpene hydrocarbons*							0.649	3.079	2.859
*Oxygenated sesquiterpenes*							7.979	31.504	15.141
*Aliphatic hydrocarbons*							26.66	4.891	10.394
*Oxygenated aliphatic hydrocarbons*							14.109	6.255	19.652
*Aromatics*							18.3	6.545	10.948
**Total identified**							**97.322**	**96.805**	**97.999**

* Components are recorded as per their order of elution from HP-5MS column; a = tentatively identified; compounds higher than 5.0% are highlighted in boldface; LRI_Exp_ = linear retention index computed with reference to the *n*-alkanes mixture (C8-C31) on HP-5MS column; LRI_Lit_ = linear retention index from the literature [23,24,27,28,29]; Hex = hexane extract of *A. judaica*; Chl = chloroform extract of *A. judaica*; MeOH = methanol extract of *A. judaica*.

As per the results given in the Table 1, oxygenated monoterpenes were present in significant amounts in all three extracts. In particular, the Hex and MeOH contained 29.0% and 39.0%, respectively, while the Chl extract exhibited the highest percentage of these components, amounting to 42.8% of the total constituents. On the other hand, the oxygenated aliphatic hydrocarbons were present at distant second position in the studied extracts, which were present in the amounts of 14.1%, 6.2%, and 19.6%, in the Hex, Chl, and MeOH extracts, respectively. Apart from these, oxygenated sesquiterpenes, aliphatic hydrocarbons, and aromatics were also present in appreciable amounts. However, there was a large difference between the amount of these components among different extracts. For instance, the Chl extract demonstrated the highest amount of oxygenated sesquiterpenes equivalent to 31.5%, whereas the Hex and MeOH contained 7.9 and 15.1% of these compounds. Similarly, with regards to aliphatic hydrocarbons, the Hex extract contained the highest amount (26.6%), which was followed by the MeOH (10.3%) and Chl (4.8%) extracts. In the case of aromatics, the trend was dominated by Hex (18.3%), which was followed by MeOH (10.9%) and Chl (6.5%) extracts. Apart from these, sesquiterpenes hydrocarbons were also present in lesser amounts, i.e., 3.0, 2.8, and 0.6% in the Chl, MeOH, and Hex extracts, respectively.

**Figure 4:**
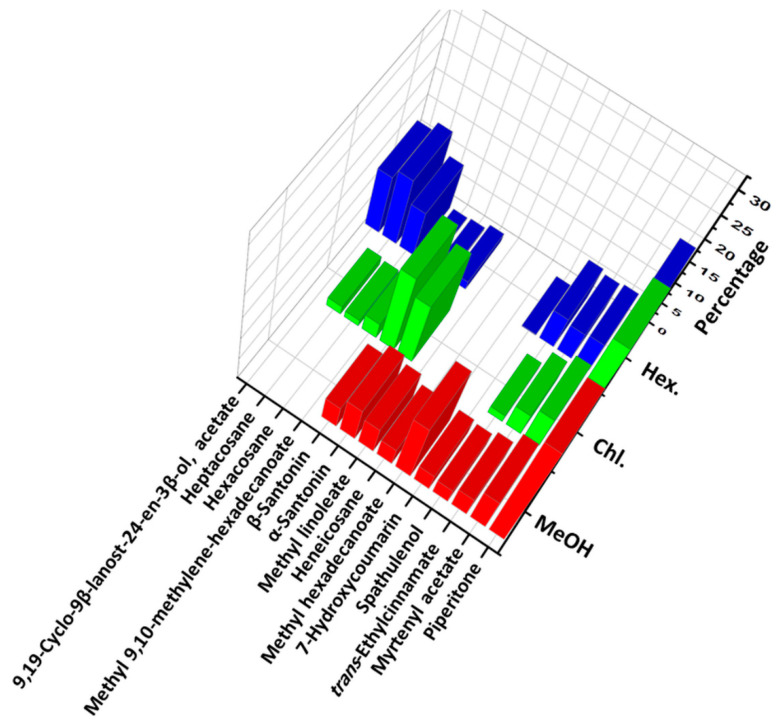
Most prominent components from CHCl_3_, methanol, and *n*-hexane extracts of *A. judaica*.

Detailed analyses of each extract revealed that the Hex extract demonstrated the presence of highest number of compounds (46), followed by Chl (18) and MeOH (17). Details of all the major components found in the three different extracts are summarized in Figure 4 and their chemical structures are given in Supplementary Materials (Figures S1–S3). Out of 46 components identified in the Hex extract, only a few compounds were present in large amounts while most of the other components existed in negligible concentrations.

From Table 1, it is evident that the Hex extract was mostly dominated by piperitone (20.2%), heptacosane (13.9), 9,19-Cyclo-9*β*-lanost-24-en-3*β*-ol, acetate (12.1%), hexacosane (9.5%), *trans*-ethylcinnamate (9.3%), spathulenol (5.0%), and myrtenyl acetate (4.2%). Among these compounds, most of the components were also present in the other two extracts, Chl and MeOH; however, their amounts vary significantly. Particularly, piperitone was present in large amounts in all three extracts and was the most dominating compound of the Chl (28.8%) and MeOH (26.1%) extracts. Apart from this, myrtenyl acetate, *trans*-ethylcinnamate, spathulenol, α-santonin, and β-santonin were also found in the three different extracts in varying quantities. On the other hand, some compounds were specifically found in only one extract, for instance, 9,19-Cyclo-9*β*-lanost-24-en-3*β*-ol, acetate (12.1%) and methyl hexadecanoate (13.5%) were specific to the Hex and MeOH extracts, respectively. Literature surveys regarding the phytoconstituents of different contents of the *A. judaica* population including essential oils, aerial parts, etc. from other countries have mostly indicated the presence of flavonoids, polyphenols, terpenes, etc. [30,31,32]. Notably, similar to the case of *A. judaica* of Saudi Arabia, piperitone is also present in significant amounts in the *A. judaica* belonging to the other regions of the world [33,34,35]. Piperitone is an oxygenated monoterpene, which is mainly responsible for the aroma of the plants and is widely used in fragrances, is mostly present in various aromatic plants such as *Eucalyptus dives*, *Micromeria fruticose*, *Mentha spicata* L., etc. [36]. Piperitone exhibits several biological properties such as insecticidal, repellent, and anti-appetent properties [37]. Indeed, in some studies, the high antimicrobial properties of the plant contents are directly attributed to the proportion of piperitone [38]. Apart from this, another compound, santonin, is distinctly present only in Chl in an excessive amount. Both α and β derivatives of santonin were found in the Chl extract in amounts of 17.1 and 13.7%, respectively, and just 7.7 and 5.0% in MeOH and 1.7 and 0.5% in the Hex. Santonin derivatives are sesquiterpene lactones, which are typically isolated from plants and possesses diverse biological properties including antibacterial, anti-inflammation, antimalaria, anticancer, etc. [39,40].

Upon comparing results of the chemical constituents of *A. judaica* in the present study with those reported from the same species in previous studies [31,33,41,42], it is significant to notice that pipertone was found to be the most versatile compound that was present as a major compound in almost all the volatile oils of *A. judaica*, except from the oil of *A. judaica* investigated from Irbid [31], where (*E*)-ethyl cinnamate was determined as the major constituent. Moreover, ethyl cinnamate was also detected in different proportions in most of the studied oil compositions of *A. judaica* [33,41] including the present study, as shown in Table 2. However, this compound was not present in the oil of *A. judaica* studied from Ilizi [42]. These variations in the chemical compositions of *A. judaica* volatile oils could be attributed to various factors including environmental and climatic conditions and geographic features [42,43].

### 3.1. Antibacterial Properties

The extracts of *A. judaica* were tested for their efficiency against Gram-positive and Gram-negative bacterial strains, while Ciprofloxacin, a prescription antibiotic, was employed as a control for the study. It was observed that the methanol extract was effective against *S. aureus* and *K. planticola,* which are Gram-positive and Gram-negative bacterial strains, respectively; however, it displayed mild activity against *M. luteus* and *E. coli* strains. Furthermore, the hexane extract and chloroform extract showed excellent antibacterial efficiency against the Gram-positive strains *S. aureus* and *M. luteus* as well as *K. planticola,* a Gram-negative strain.

From the results obtained, it is observed that the methanol extract displays significant activity against *S. aureus* and *K. planticola* bacterial strains with 3.9 µg/mL and 1.9 µg/mL, respectively, but very mild activity against *M. luteus* and *E. coli* (Table 3). Moreover*,* the extracts obtained from hexane and chloroform are highly active against the tested Gram-positive bacterial strains and *K. planticola,* a Gram-negative bacterial strain. The MIC values obtained against these strains are similar to the control used, i.e., Ciprofloxacin, a prescription antibiotic. While all the extracts, i.e., the hexane, chloroform, and methanol, display mild anti-bacterial activity against the bacterial strain *E. coli*, it is important to mention here that hexane and chloroform extracts could play a potential role in the development of efficient antibacterial agents in future studies. These two extracts could be recommended for the isolation and identification of an active antibacterial agent from *A. judaica*.

### 3.2. Anticancer Properties

**Figure 5:**
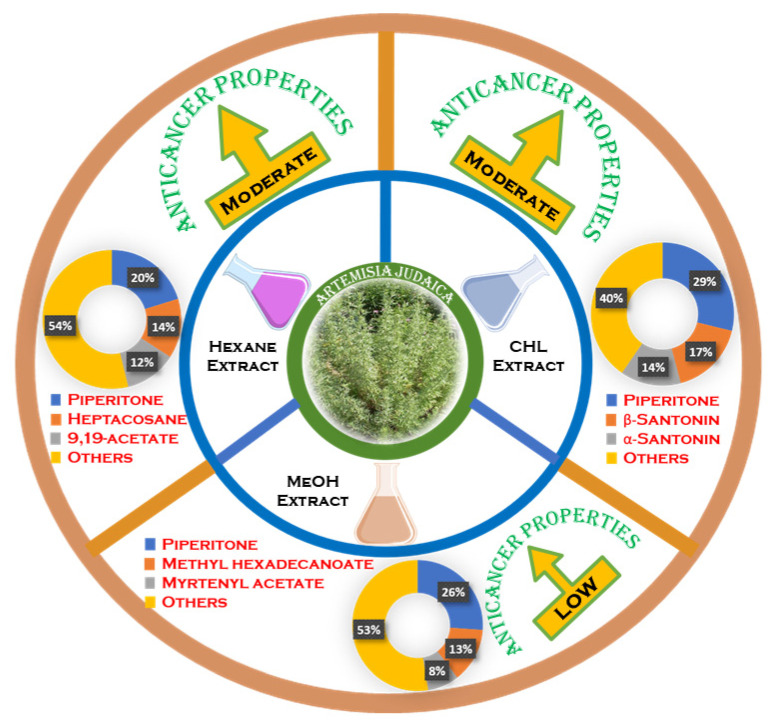
Anticancer activity variations of CHCl_3_, MeOH, and hexane extracts of *A. judaica*.

In addition to the antibacterial studies, the isolated extracts of *A. judaica* were also tested for their efficiency against various cancer cell lines, such as hepatic cancer cells (HepG2), prostate cancer cells (DU145), cervical cancer cells (Hela), and human lung cancer cells (A549), while Doxorubicin, a prescription anticancer drug, was employed as a control for the study (Table 4). All the extracts showed different levels of activity, and the variations in anticancer activity of the CHCl_3_, methanol, and *n*-hexane extracts of *A. judaica* are postulated in Figure 5.

From Table 3, it is evident that all the tested extracts display mild to moderate anticancer activity, with the best IC_50_ value of 35.41 ± 1.78 µg/mL obtained for the chloroform extract against the DU145 cancer cell line, i.e., the prostate cancer cell line. This activity was comparable to that of the hexane extract as well, for which the IC_50_ value was 48.49 ± 0.16. On the other hand, the lowest activity was found for the methanol extract of *A. judaica* against the A549 cell line with an IC_50_ value of 168.54 ± 5.13 µg/mL. The methanol extract also showed lower activity against the other tested cell lines in comparison to those of the hexane and chloroform extracts. Moreover, careful observation of Table 3 suggests that the hexane extract of *A. judaica* possessed higher activity against HepG2, Hela, and A549 cancer cell lines compared to those of the chloroform and methanol extracts. Therefore, hexane extract of *A. judaica* could be considered for further studies to isolate active ingredients for the development of novel anticancer molecules.

It is worth mentioning here that there are no prior reports on the comparative study of anticancer activity of *A. judaica* extracts obtained from solvents of varying polarities. However, there are some studies which report the anticancer activity of *A. judaica* extracts using polar solvents such as methanol [17,44,45], unlike the study reported in our manuscript wherein we employed two solvents, i.e., hexane and chloroform, prior to methanol. On comparing anticancer activity results of our methanolic extract with those reported earlier [17,44,45], it was found that the methanolic extract in this study showed mild anticancer activity compared to that reported in previous studies. This might be due to the partition of the active ingredients of *A. judaica* into hexane and chloroform extracts during the extraction process, as the hexane and chloroform extracts in the present study have also shown significant anticancer activity similar to those reported earlier [17,44,45].

## 4. Conclusions

Herein, to determine the effect of extraction solvents on the content of secondary metabolites, antimicrobial and anticancer properties were evaluated for three different extracts (Hex, Chl, and MeOH) of *A. judaica* grown in Saudi Arabia. All three different extracts of the aerial parts of *A. judaica* exhibited important disparities in their chemical compositions, and variations in amounts of some lead phytoconstituents were also noticed. In this study, the investigated plant extracts displayed piperitone as the major component, which was present in varied amounts in the different extracts. Among all three different extracts, the Chl extract of *A. judaica* showed superior antimicrobial and anticancer properties, which could be ascribed to the distinct presence of the large amounts of piperitone (28.8%) and santonin (α =17.1%, β = 13.7%), which are known to demonstrate excellent biological properties. These results offer scientific evidence of the medicinal properties of *A. judaica* in traditional medicine. *A. judaica* extracts can prove to be useful resources for the development of plant-based pharmaceuticals, functional foods, and other cosmetic products. However, a detailed biological activity-guided chromatographic analysis is necessary to extract potentially active phytoconstituents from these extracts.

## Figures and Tables

**Table 2:** Most dominating compounds of *A. judaica* investigated from different parts of the world.

S. No.	Country	City	Major Components (%)	Reference
1.	Jordan	Irbid	(*E*)-Ethyl cinnamate (21.46), artemisia ketone (20.76), davanone (16.78), (*Z*)-ethyl cinnamate (12.13), yomogi alcohol (5.15), artemisyl acetate (4.70), and chrysanthenone (4.60).	[31]
		Al-Mudawarh	Piperitone (30.4), camphor (16.1) and ethyl cinnamate (11.0) and chrysanthenone (6.7) and piperitenone oxide (3.9).	[33]
2.	Algeria	Tassili n’Ajjer	Piperitone (71.1), 3-methyl-ethylbutanoate (12.3) and 1-butanol (3.5).	[41]
		Ilizi	Piperitone (61.9), terpinen-4-ol (4.6) and bornyl acetate (3.0).	[42]
3.	Saudi Arabia	Madinah	Piperitone (20–29), myrtenyl acetate (6.7–8.0), *α*-santonin (1.7–14.0), *β*-santonin (0.5–17%) and *trans*-ethyl cinnamate (4.6–6.3), methyl hexadecanoate (0–13.5), 9,19-cyclo-9*β*-lanost-24-en-3*β*-ol, acetate (0–12.1), heptacosane (0–14) and hexacosane (0–10).	Present study

**Table 3:** Antimicrobial activity of various extracts of *A. judaica* grown in Saudi Arabia against Gram-positive and Gram-negative bacteria.

Tested Extracts of *A. judaica*	Minimum Inhibitory Concentration (µg/mL)			
	Gram-Positive		Gram-Negative	
	*S. aureus* MTCC 96	*M. luteus* MTCC 2470	*K. planticola* MTCC 530	*E. coli* MTCC 739
MeOH	3.9	>250	1.9	>250
Hex	0.9	0.9	0.9	>250
Chl	0.9	0.9	0.9	>250
Ciprofloxacin *	0.9	0.9	0.9	0.9

*—Positive control.

**Table 4:** Anticancer activity of various extracts of *A. judaica* grown in Saudi Arabia against various cancer cell lines.

Tested Extracts of *A. judaica*	IC_50_ (µg/mL)			
	HepG2	DU145	Hela	A549
**MeOH**	99.95 ± 4.13	51.97 ± 0.19	67.12 ± 1.75	168.54 ± 5.13
**Hex**	54.30 ± 0.66	48.49 ± 0.16	54.40 ± 1.11	67.36 ± 0.41
**Chl**	56.89 ± 0.37	35.41 ± 1.78	61.85 ± 0.18	76.48 ± 4.7
Doxorubicin	0.72 ± 0.012 (µM)	0.36 ± 0.01 (µM)	0.8 ± 0.71 (µM)	0.55 ± 0.16 (µM)

Results are expressed as mean ± SD.

## Data Availability

Data contained within the article and Supplementary Materials.

## Supplementary Materials

The following supporting information can be downloaded at: https://www.mdpi.com/article/10.3390/life12111885/s1, Scheme S1: Gas Chromatography (GC) and Gas Chromatography−Mass Spectrometry (GC-MS) Analysis of Essential Oils; Scheme S2: Linear retention indices (LRIs); Scheme S3: Identification of volatile components; Figure S1: Chemical structure of major components identified from hexane extracts of *A. judaica*; Figure S2: Chemical structure of major components identified from CHCl_3_ extracts of *A. judaica.* Figure S3: Chemical structure of major components identified from methanol extracts of *A. judaica.*
